# Captivity-driven microbiota reshaping: A cross-species analysis of divergent patterns in the gut microbiota of giant pandas (*Ailuropoda melanoleuca*), red pandas (*Ailurus fulgens*), and Asiatic black bears (*Ursus thibetanus*)

**DOI:** 10.1371/journal.pone.0332481

**Published:** 2025-10-08

**Authors:** Lin Xu, Zhengquan Hu, Xin Zhang, Siyuan Zhang, Wenjing Li, Minglei Wang, Ruihong Ning, Wei Guo

**Affiliations:** 1 China Conservation and Research Center for the Giant Panda, Key Laboratory of State Forestry and Grassland Administration on the Giant Panda, Chengdu, China; 2 Sichuan Provincial Engineering Laboratory for Prevention and Control Technology of Veterinary Drug Residue in Animal-Origin Food, Chengdu Medical College, Chengdu, China; 3 Sichuan Wolong National Nature Reserve Administration, Wolong, Sichuan Province, China; 4 Clinical IVD Joint Research Center of Chengdu Medical College-Maccura Biotechnology, Chengdu, China; Concordia University Irvine, UNITED STATES OF AMERICA

## Abstract

The relative influence of diet, host phylogeny, and environment on animal gut microbiota remains unresolved, particularly for endangered ursids lacking quantitative data. Here, we systematically evaluated the drivers of gut microbial assembly in captive versus wild giant pandas (*Ailuropoda melanoleuca*), red pandas (*Ailurus fulgens*), and Asiatic black bears (*Ursus thibetanus*) using 16S rRNA V4 sequencing. Compared with wild cohorts, captive giant pandas exhibited significantly reduced α-diversity (*P* < 0.05), whereas captive red pandas and black bears showed significant increases (*P* < 0.05). Weighted UniFrac-based β-diversity analysis revealed that intra-species distances between captive and wild individuals exceeded those observed between species within either habitat (*P* < 0.001), indicating profound community restructuring under captivity. At the phylum level, captive animals were dominated by Firmicutes (68.6 ± 23.0%), in contrast to Proteobacteria dominance in wild populations (81.2 ± 17.6%). Genus‐level shifts included an enrichment of *Sarcina* in captive bears and *Streptococcus* and *Escherichia–Shigella* in captive pandas, whereas wild bears and pandas were predominantly enriched in *Burkholderia* and *Pseudomonas*, respectively. PERMANOVA attributed 21.6% of community variance to environment (*F = *23.62), compared to 12.3% for host phylogeny (*F* = 6.75) and 3.9% for diet (*F* = 4.32). These findings demonstrate that captive management is the primary determinant of gut microbiota divergence in giant pandas, red pandas, and Asiatic black bear provide microbiome-based guidance for improving captive husbandry and reintroduction success.

## Introduction

Gut microbiota, regarded as the “second genome” of animals, plays pivotal roles in host nutrient metabolism [1], immune modulation [2], and environmental adaptation [3]. Diet is one of the most immediate determinants of gut microbial composition, with herbivores, omnivores, and carnivores each establishing distinct microbial community structures [4]. Phylogenetically related hosts may exhibit convergent gut microbial communities under stable ecological conditions, though transient perturbations can obscure this signal. For example, baleen whales, which are marine carnivores descended from ruminant-like terrestrial ancestors such as cattle and hippos, retain gut microbial compositions and functional profiles that closely resemble those of their herbivorous relatives. [5]. In recent years, the perturbative effects of captivity on wildlife gut microbiota have garnered increasing attention [6,7]; factors such as antibiotic and drug exposure, human interaction, and husbandry practices can substantially reshape host microbial communities. Nevertheless, the relative contributions of diet, host genetics, and captive environment to gut microbiota remodeling remain poorly resolved.

The giant panda (*Ailuropoda melanoleuca*), although a member of *Ursidae*, is a specialized bamboo feeder. Its sympatric counterpart, the red panda (*Ailurus fulgens*), phylogenetically belongs to *Procyonidae* yet similarly depends on bamboo (> 90% of its diet). In contrast, the giant panda’s closest extant relative, the Asiatic black bear (*Ursus thibetanus*), retains the typical ursid omnivorous feeding habit. However, all three species overlap in wild habitats and are managed under similar captive conditions (e.g., bamboo-based diets, enclosures). This controlled for confounding environmental variables, enabling clearer isolation of captivity effects. Occupy overlapping habitats yet exhibit highly divergent dietary specializations (bamboo versus omnivore), providing a natural comparative model to disentangle the relative roles of dietary niche, host phylogeny, and environmental factors in gut microbiome assembly. Whether bamboo specialization has driven convergent turnover of gut microbiota across these taxonomically distinct species remains debated: some studies report greater similarity between panda microbiota than with other bears [8], whereas others demonstrate that giant pandas maintain core ursid microbial taxa and functions distinct from other bear species [9–11]. Notably, captivity has been shown to significantly alter α-diversity and community structure in giant pandas, red pandas, and other ursids [6,12,13]. However, it remains unclear whether the microbiota responses to captivity stress share cross-species commonalities among endangered ursids, a gap that hinders the development of targeted microbiome-based conservation strategies.

Here, we present the first cross-species, quantitative analysis of gut microbiota divergence in three endangered ursids under captive versus wild conditions using high-throughput sequencing of the 16S rRNA V4 region. Our findings aim to provide critical microbiome‐based insights to optimize captive management practices and improve reintroduction success for giant pandas and related endangered bear species.

## Materials and methods

### Sample collection

A stratified sampling design was employed to obtain fecal samples from both captive and wild populations. Captive specimens were collected as follows: giant pandas (*Ailuropoda melanoleuca*, n = 10) from the China Conservation and Research Center for the Giant Panda (Ya’an, China); red pandas (Ailurus fulgens, n = eight) and Asiatic black bears (Ursus thibetanus, n = six) from Bifengxia Ecological Zoo. Wild specimens comprised: giant pandas (n = 16) and red pandas (n = 16) from the Fengtongzhai National Nature Reserve (Ya’an, China); Asiatic black bears from two reserves: Fengtongzhai (n = nine) and Tangjiahe National Nature Reserve (Guangyuan, China, n = eight).

Fecal samples were collected following a strict spatial and temporal protocol to ensure sampling independence and avoid collecting multiple samples from the same individual, we applied the following criteria: For pandas, fecal samples were collected from defecation events occurring within 72-hour intervals and located beyond the species’ home-range diameter (giant panda: 5 km; red panda: 1.5 km) [14,15]; For black bears, samples were collected within a 24-hour window due to their large home ranges (>100 km), which precluded spatial separation criteria. This conservative approach leverages the species’ sedentary behavior to maximize the probability that each sample represents a unique individual. All fecal samples were collected during summer months (June-August) to control for potential seasonal variations in gut microbiota composition. Fecal samples were collected aseptically, immediately flash-frozen in liquid nitrogen, and stored at –80°C until processing. Captive individuals were included only if they had not received antibiotic treatment within one month prior to sampling.

### Total microbial DNA extraction

Under sterile conditions, the exterior of each frozen fecal pellet was trimmed to remove potential contaminants. Approximately 200 mg (± 5 mg) of the inner core was weighed into pre-chilled tubes. Genomic DNA was extracted using the PowerFecal DNA Isolation Kit (Qiagen, Germany) following the manufacturer’s protocol. DNA concentration and purity were quantified with a Qubit™ 2.0 Fluorometer (Thermo Fisher Scientific, USA), and integrity was assessed by agarose gel electrophoresis. Only DNA samples meeting the following criteria were used for library preparation: (1) concentration > 10 ng/µL; (2) total yield > 100 ng; (3) high integrity (single band > 20 kb) with A₂₆₀/A₂₃₀ ratio > 2.0.

### 16S rRNA V4 region sequencing

The V4 hypervariable region of the bacterial 16S rRNA gene was amplified using barcoded primer pair 515F (5’-GTGCCAGCMGCCGCGGTAA-3’) and 806R (5’-GGACTACHVGGGTWTCTAAT-3’). PCR reactions (25 µL) contained Phusion® High-Fidelity PCR Master Mix (15 µL; New England Biolabs), each primer at 0.2 µM, and 10 ng of template DNA. Thermocycling conditions were: initial denaturation at 98°C for 1 min; 30 cycles of 98°C for 10 s, 50°C for 30 s, and 72°C for 30 s; and a final extension at 72°C for 5 min. Equimolar PCR products were pooled and purified using the QIAquick Gel Extraction Kit (Qiagen, Germany). Sequencing libraries were constructed with the TruSeq® DNA PCR-Free Library Prep Kit (Illumina, USA) following the manufacturer’s instructions (end repair/A-tailing, adapter ligation with dual indices). Library quality and concentration were evaluated on a Qubit™ 2.0 Fluorometer and an Agilent 2100 Bioanalyzer. Qualified libraries were normalized and sequenced on an Illumina NovaSeq 6000 platform (2 × 250 bp paired-end) by Novogene Co., Ltd.

### Bioinformatic processing

Raw sequence data were processed using QIIME 2 (v.2020.6) [16]. DADA2 was employed for quality control and sequence inference, including: (i) trimming of low-quality 3′ ends and removal of primer/adapters; (ii) denoising to distinguish true biological sequences from errors; (iii) merging of paired-end reads; and (iv) filtering out bases with Phred scores < 20. Chimeric sequences were further removed using USEARCH 8 [17], yielding high-quality “clean reads.” Clean reads were dereplicated into amplicon sequence variants (ASVs) via DADA2’s denoising algorithm. Representative ASV sequences were taxonomically assigned with BLAST+ against the SILVA 1.3.2 database at ≥ 97% identity [18,19]. ASVs present in > 50% of samples per group were designated as core ASVs. α-Diversity metrics (Shannon index, observed ASVs) were calculated, and β-diversity was assessed by principal coordinates analysis (PCoA) on weighted UniFrac distances to evaluate community similarity and inter-group differences.

### Statistical analysis

Statistical analyses were performed in SPSS 23.0. Data normality and homogeneity of variance were assessed; two-group comparisons of normally distributed data with equal variances used independent-samples t-tests, while non-normal data were compared by Kruskal–Wallis tests. For comparisons among three or more groups meeting normality and homogeneity criteria, one-way ANOVA was applied, followed by Duncan’s post hoc test when overall differences were significant (P < 0.05). Permutational multivariate analysis of variance (PERMANOVA) was conducted in R (vegan package) [20] to quantify the effects of diet, host species, and environment on community structure. A random forest classifier (randomForest package; proximity = TRUE, ntree = 1000, mtry = 22) [21] identified key taxa distinguishing captive from wild individuals. All figures were generated using ggplot2 in R [22].

### Ethical approval and permits

This study was approved by the Institutional Animal Care and Use Committee of the China Conservation and Research Center for the Giant Panda (Approval No. CCRCGP2022003). All fieldwork and data collection were conducted in full compliance with relevant regulations and guidelines.

## Results

### Core ASVs composition in giant pandas, red pandas, and Asiatic black bears across different habitats

**Fig 1:**
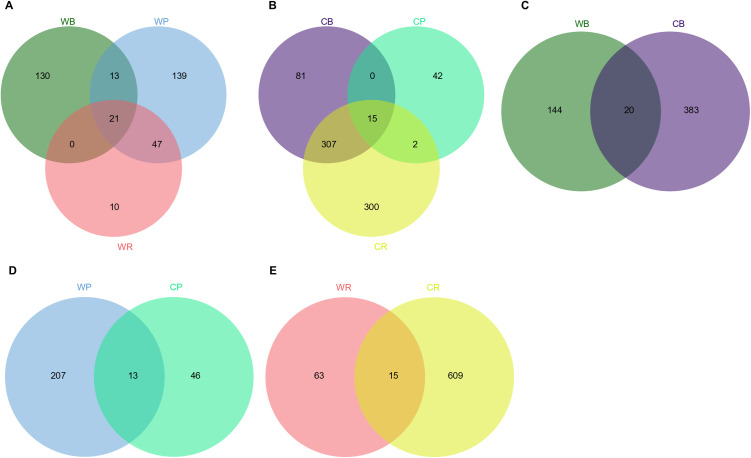
Venn Diagram Analysis of Core Amplicon Sequence Variants (ASVs) in Gut Microbiota of Giant Pandas, Red pandas and Asiatic Black Bears Across Different Habitats. **(A)** Comparative analysis of core ASVs among wild giant pandas (WP), wild red pandas (WR) and wild Asiatic black bears (WB). **(B)** Comparative analysis among captive giant pandas (CP), captive red pandas (CR) and captive Asiatic black bears (CB). **(C)** Core ASV comparison between wild (WB) and captive (CB) Asiatic black bears. **(D)** Core ASV comparison between wild (WP) and captive (CP) giant pandas. **(E)** Core ASV comparison between wild (WR) and captive (CR) red pandas.

We identified pronounced differences and overlaps in the core ASV assemblages of giant pandas, red pandas, and Asiatic black bears when comparing wild and captive cohorts. In wild populations (Fig. 1A), giant pandas harbored 139 unique ASVs, red pandas 130, and black bears 10, with 21 ASVs (5.8%) shared among all three species. Pairwise overlaps included 34 ASVs (9.4%) between giant pandas and black bears, 68 ASVs (18.9%) between giant pandas and red pandas, and 21 ASVs (5.8%) between black bears and red pandas. In contrast, captive groups (Fig. 1B) exhibited fewer species-specific ASVs—42 in giant pandas, 300 in red pandas, and 81 in black bears, and only 15 ASVs (2.0%) were common to all three. Notably, captive giant pandas and black bears shared 15 ASVs (2.0%), captive giant pandas and red pandas shared 17 ASVs (2.3%), while captive black bears and red pandas shared 322 ASVs (43.1%). Cross-habitat comparisons further revealed that wild and captive black bears shared 20 ASVs (3.7%; Fig. 1C), wild and captive giant pandas shared 13 ASVs (4.9%; Fig. 1D), and wild and captive red pandas shared 15 ASVs (2.2%; Fig. 1E), underscoring the significant impact of captivity on core gut microbiota composition.

### Comparison of α-diversity in gut microbiota across habitats

**Fig 2:**
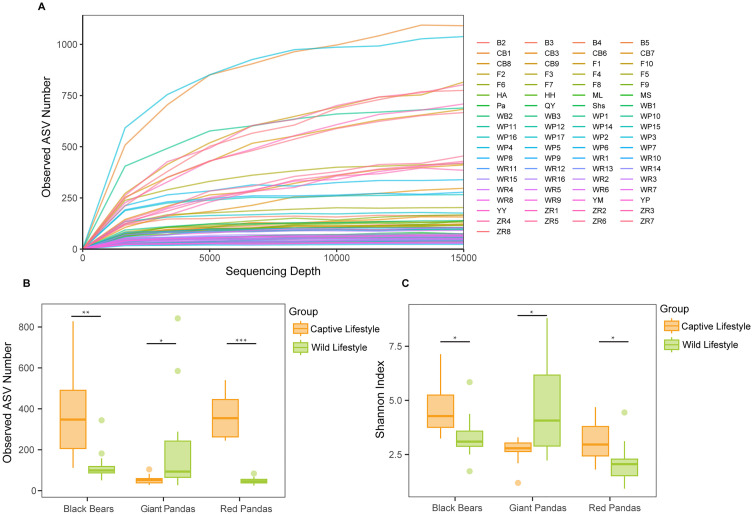
Comparative Analysis of Gut Microbiota Alpha Diversity in Giant Pandas, Red Pandas, and Asiatic Black Bears Across Habitats. **(A)** Rarefaction curves of microbial alpha diversity for all fecal samples. **(B)** Comparison of Observed ASVs between wild and captive groups. **(C)** Comparison of Shannon diversity index between wild and captive groups. *P < 0.05, **P < 0.01, ***P < 0.001 (Kruskal-Wallis test); Boxplots show median (central line), interquartile range (box), and data range (solid lines).

To assess the adequacy of sequencing coverage for diversity estimation, we generated rarefaction curves by subsampling 15,000 reads per sample (Fig. 2A). The curves approached an asymptote with increasing sequencing depth, indicating that the sampling effort was sufficient to capture the majority of microbial diversity. In α-diversity comparisons, captive Asiatic black bears and red pandas exhibited significantly higher observed ASV counts and Shannon diversity indices than their wild counterparts (**P* *< 0.05). Conversely, captive giant pandas showed significantly lower observed ASVs and Shannon indices compared to wild giant pandas (*P* < 0.05) (Figs. 2B and 2C).

### Comparison of β-diversity in gut microbiota across habitats

**Fig 3:**
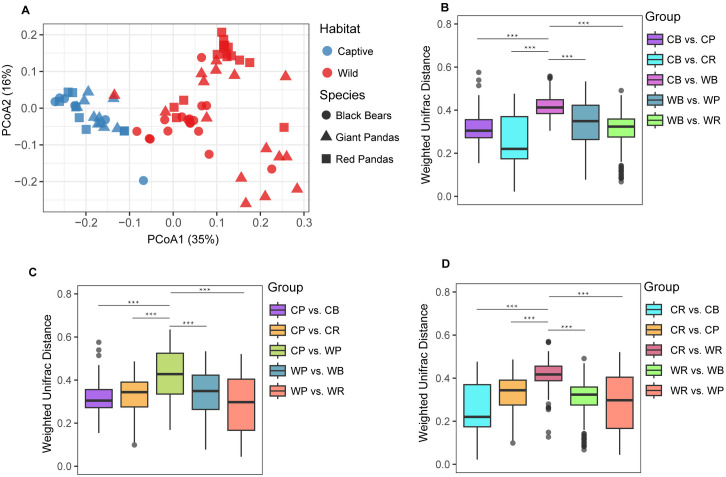
Comparative Analysis of Gut Microbiota Beta Diversity in Giant Pandas, Red Pandas, and Asiatic Black Bears Across Habitats. **(A)** Principal coordinate analysis (PCoA) based on weighted UniFrac distance matrix, with point shapes indicating species and colors denoting habitat types. **(B)** Intra-/inter-group comparisons for Asiatic black bears: Wild (WB) vs. captive (CB) individuals and cross-species comparisons. **(C)** Intra-/inter-group comparisons for giant pandas: Wild (WP) vs. captive (CP) individuals and cross-species comparisons. **(D)** Intra-/inter-group comparisons for red pandas: Wild (WR) vs. captive (CR) individuals and cross-species comparisons. *P < 0.05, **P < 0.01, ***P < 0.001 (one-way ANOVA with Duncan’s post hoc test).

Principal Coordinates Analysis (PCoA) based on weighted UniFrac distance matrices revealed that habitat exerts a significant influence on the gut microbiota structure of all three ursid species (Fig 3A). Specifically, wild giant pandas, red pandas, and Asiatic black bears did not differ significantly in β-diversity, indicating highly similar community composition within the same wild environment; a comparable pattern was observed under captive conditions (Fig 3A). However, within‐species comparisons between wild and captive cohorts (i.e., CB vs. WB, CP vs. WP, CR vs. WR) demonstrated that weighted UniFrac distances were significantly greater than those observed between different species within the same habitat (*P* < 0.05; Figs. 3B–D), indicating that habitat transition impacts the gut microbiota of a single species more profoundly than inherent interspecific differences.

### Comparison of phylum- and genus-level community composition across habitats

**Fig 4:**
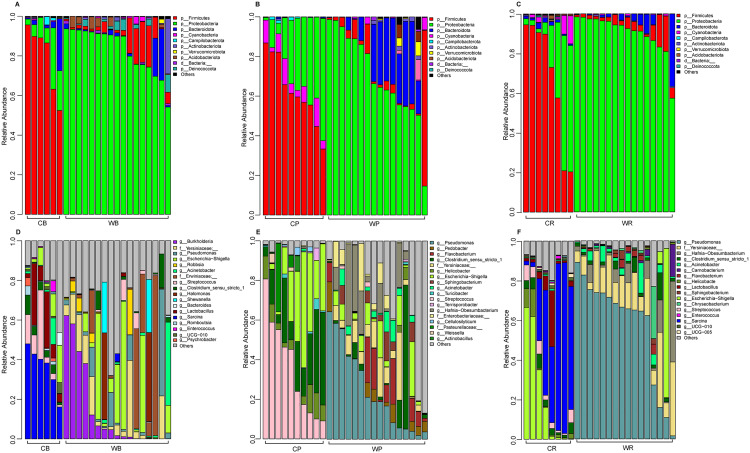
Comparative Analysis of Gut Microbiota Composition in Giant Pandas, Red Pandas, and Asiatic Black Bears Across Habitats. **(A)** Top 10 phyla comparison between wild (WB) and captive (CB) Asiatic black bears. **(B)** Top 10 phyla comparison between wild (WP) and captive (CP) giant pandas. **(C)** Top 10 phyla comparison between wild (WR) and captive (CR) red pandas. **(D)** Top 10 genera comparison between wild (WB) and captive (CB) Asiatic black bears. **(E)** Top 10 genera comparison between wild (WP) and captive (CP) giant pandas. **(F)** Top 10 genera comparison between wild (WR) and captive (CR) red pandas. Error bars represent standard deviation, with color gradients indicating taxonomic units.

At the phylum level, captive and wild cohorts displayed marked differences: captive Asiatic black bears, giant pandas, and red pandas were all dominated by Firmicutes, with mean relative abundances of 79.45 ± 17.36% (Fig. 4A), 62.85 ± 17.13% (Fig. 4B), and 67.60 ± 31.62% (Fig. 4C), respectively, whereas their wild counterparts were overwhelmingly dominated by Proteobacteria at 83.20 ± 11.77%, 70.10 ± 22.67%, and 90.26 ± 10.26%, respectively. At the genus level, distinct community signatures were also evident between habitats. In Asiatic black bears, captive individuals were primarily enriched in *Sarcina* (35.90 ± 11.36%) and *Streptococcus* (11.03 ± 12.77%), whereas wild bears were dominated by *Burkholderia* (13.76 ± 11.84%) and the *Escherichia–Shigella* complex (13.77 ± 11.53%) (Fig. 4D). In giant pandas, captivity favored *Streptococcus* (35.99 ± 24.71%) and *Escherichia–Shigella* (23.01 ± 19.18%), while wild pandas harbored a higher proportion of *Pseudomonas* (24.77 ± 21.63%) (Fig. 4E). Similarly, red pandas in captivity exhibited elevated levels of *Sarcina* (44.96 ± 32.77%) and *Escherichia–Shigella* (22.86 ± 18.23%), in contrast to wild red pandas, which were dominated by *Pseudomonas* (57.14 ± 27.12%) (Fig. 4F).

### Relative contributions of diet, host phylogeny, and environment to gut microbiota structure in giant pandas, red pandas, and Asiatic black bears

Permutational multivariate analysis of variance (PERMANOVA) revealed that environment, diet, and host phylogeny each significantly influenced the gut microbial community structure of giant pandas, red pandas, and Asiatic black bears (*P* < 0.0001; Table 1). Among these factors, environment explained the greatest proportion of variance (R² = 21.58%, *F* = 23.62), followed by host phylogeny (R² = 12.34%, **F* *= 6.75), while diet accounted for the smallest share of variance (R² = 3.94%, *F* = 4.32).

**Table 1:** Assessment of key factors influencing beta diversity using permutational multivariate analysis of variance (PERMANOVA, Adonis algorithm).

Factors	Variables (Groups)	Bray–Curtis	
		pseudo-F	*p*-value
Environment	Wild/Captive	23.62	< 0.001
Diet	Bamboo/Omnivorous	6.75	< 0.001
Host	*Ursidae/ Procyonidae*	4.32	< 0.001

### Identification of cross-species gut microbial biomarkers distinguishing captive and wild populations

**Fig 5:**
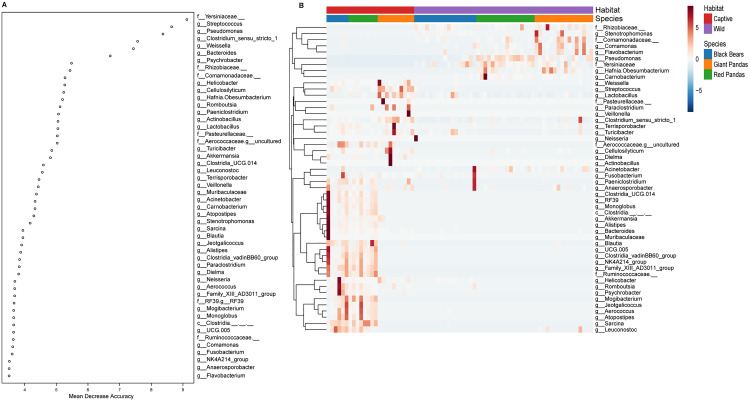
Identification of Gut Microbiota Biomarker Genera Distinguishing Wild and Captive Animals (A) Top 50 discriminative genera selected by random forest classifier, ranked by mean decrease accuracy. **(B)** Heatmap visualization of Z-score normalized relative abundance for the top 50 biomarker genera, with row-wise clustering using Ward’s hierarchical method.

Using a random forest classifier, we identified key genus-level biomarkers that discriminate between captive and wild individuals across the three ursid species (Fig. 5A). The top 50 discriminatory genera by identified based on their Mean Decrease in Accuracy (MDA) scores included *Yersiniaceae*, *Streptococcus*, *Pseudomonas*, *Clostridium sensu stricto 1*, *Weissella*, and *Bacteroides*. Among these, *Rhizobiaceae*, *Stenotrophomonas*, *Comamonadaceae*, *Comamonas*, *Flavobacterium*, *Pseudomonas*, *Yersiniaceae*, *Obesumbacterium*, and *Carnobacterium* were significantly enriched in wild individuals. In contrast, the majority of discriminative taxa—including *Sarcina*, *Streptococcus*, *Weissella*, *Clostridium sensu stricto 1*, *Bacteroides*, *Psychrobacter*, members of *Aerococcaceae*, *Lactobacillus*, and *Blautia*—were markedly more abundant in captive animals (Fig. 5B). Notably, captive Asiatic black bears and red pandas shared a more similar biomarker profile, whereas wild giant pandas and wild red pandas exhibited closer alignment in their dominant genera.

## Discussion

This study provides the first cross‐species evidence that captivity exerts a dominant reshaping effect on the gut microbiota of endangered ursids and quantitatively ranks the relative contributions of environment, host phylogeny, and diet. Our findings demonstrate that, compared to host genetics and dietary specialization, the artificial captive environment is the primary driver of microbiota divergence in giant pandas, red pandas, and Asiatic black bears. This insight advances our understanding of microbial responses to captivity stress and carries important implications for refining conservation practices.

We observed species‐specific effects of captivity on α‐diversity. Consistent with previous reports, captive giant pandas exhibited a significant reduction in both observed ASVs and Shannon diversity [6]. In contrast, captive red pandas and black bears showed significant increases in α‐diversity, diverging from the general trend in most captive mammals, which typically experience declines in microbial richness and evenness [23–25]. We attribute this discrepancy to differences in dietary adaptation strategies: as obligate bamboo specialists, wild giant pandas rely on high-fiber bamboo to sustain a stable community of cellulose-degrading microbes, whereas diets in captivity, reduced in bamboo and enriched with refined carbohydrates (e.g., Steamed cornbread: 600–1200 g/day), likely disrupt this functional guild, leading to lower diversity. Access to diverse bamboo species and environmental microbiota in wild giant pandas enhances their gut microbial diversity. Conversely, the omnivorous red pandas and black bears, facultative bamboo consumers, may derive benefits from the more diverse captive diets (e.g., fruits, grains), which promote microbial expansion and drive community convergence in captivity.

The β‐diversity analyses revealed that captivity markedly homogenizes gut communities across species, mirroring findings in captive primates where distinct wild microbiota converge under standardized husbandry [26]. Unified management practices-diet composition, antibiotic protocols, and restricted habitat complexity-appear to suppress the shaping forces of host phylogeny and diet, producing a “microbial homogenization” effect.

The phylum‐level shift from Proteobacteria dominance in wild bears to Firmicutes enrichment in captivity underscores how environmental pressures modulate microbial metabolic potential. Firmicutes, often associated with the fermentation of readily available carbohydrates [27,28], likely flourish on starch- and sugar-rich captive diets, whereas Proteobacteria may thrive in the more varied wild milieu, aiding complex polysaccharide degradation. Notably, the enrichment of opportunistic genera such as *Streptococcus* and *Escherichia–Shigella* in captive animals suggests elevated inflammation and infection risk. In contrast, wild individuals, which are enrichment in *Burkholderia* and *Pseudomonas*(lignocellulose-degrading genera [29]), may metabolize bamboo lignin more efficiently. This metabolic capacity diminishes in captivity, potentially compromising reintroduction success.

PERMANOVA confirmed that the environment accounts for 21.58% of the microbiota variance, far exceeding the contributions of host phylogeny (12.34%) and diet (3.94%). This challenges the conventional paradigm that dietary niche or host lineage predominantly shapes gut communities. [4]. Captivity thus exerts an “override” effect, suggesting that personalized microbiome management (e.g., transplantation of wild‐type functional taxa) may be necessary to restore species‐specific microbiota prior to rewilding [30,31]. Furthermore, the genus‐level biomarkers identified by random forest classification (e.g., *Yersiniaceae*, *Streptococcus*, *Pseudomonas*) offer candidate indicators for monitoring the microbial readiness of captive pandas and bears for release [32].

Our results carry critical warnings for reintroduction programs: the loss of wild‐associated functional taxa and proliferation of opportunists in captivity may undermine host fitness post-release. For instance, while Firmicutes expansion may enhance caloric extraction, overrepresentation could impair fiber degradation, reducing giant pandas’ ability to exploit bamboo in the wild. Therefore, we recommend a dual approach: (1) mimic natural diets by increasing bamboo diversity and reducing refined carbohydrates to preserve host‐specific microbiome functions; and (2) limit antibiotic use and consider targeted probiotic or fecal microbial transplants to reestablish wild‐like microbiota. Additionally, to prevent cross‐contamination and homogenization, mixed‐species housing should be avoided.

Limitations of this study encompass restricted sample sizes (notably wild black bears) and 16S rRNA gene profiling’s inability to resolve functional potential, it underscores the critical need for metagenomic and culturomic analyses to uncover the functional capacity. However, the findings presented here provide the essential foundation and rationale for that deeper functional investigation.

Our ongoing follow-up study, employing shotgun metagenomics, is specifically designed to explore the metabolic potential and functional adaptations of these bacterial communities, particularly in relation to host diet and captivity status. Integrating metagenomics with metabolomics in future studies will clarify metabolic pathways affected by captivity, whereas detailed nutritional records will quantify diet–microbiota interactions. By examining links between microbiome shifts and host health metrics (e.g., immunity, reproduction), we can develop targeted interventions to improve endangered ursid conservation and welfare.

## Conclusion

Through cross‐species comparison, this study demonstrates that standardized captive management profoundly reshapes the gut microbiota of endangered ursids, exerting a greater effect than host phylogeny or dietary specialization and driving community convergence and functional imbalance. These findings underscore the necessity of incorporating microbial “environmental fidelity” into captive management protocols for endangered species. Strategies such as diet mimicry and targeted microbiota transplantation should be employed to reestablish wild‐type microbial community attributes, thereby enhancing the ecological resilience and reintroduction success of captive individuals.
